# What Makes Community Engagement Effective?: Lessons from the *Eliminate Dengue* Program in Queensland Australia

**DOI:** 10.1371/journal.pntd.0003713

**Published:** 2015-04-13

**Authors:** Pamela A. Kolopack, Janet A. Parsons, James V. Lavery

**Affiliations:** 1 Centre for Ethical, Social, and Cultural Risk, St. Michael’s Hospital, Toronto, Ontario, Canada; 2 Li Ka Shing Knowledge Institute of St. Michael’s Hospital, Toronto, Ontario, Canada; 3 Dalla Lana School of Public Health, University of Toronto, Toronto, Ontario, Canada; 4 Applied Health Research Centre, St. Michael’s Hospital, Toronto, Ontario, Canada; 5 Department of Physical Therapy, University of Toronto, Toronto, Ontario, Canada; Common Heritage Foundation, NIGERIA

## Abstract

**Background:**

Worldwide, more than 40% of the population is at risk from dengue and recent estimates suggest that up to 390 million dengue infections are acquired every year. The Eliminate Dengue (ED) Program is investigating the use of *Wolbachia*-infected, transmission-compromised, mosquitoes to reduce dengue transmission. Previous introductions of genetically-modified strategies for dengue vector control have generated controversy internationally by inadequately engaging host communities. Community Engagement (CE) was a key component of the ED Program’s initial open release trials in Queensland Australia. Their approach to CE was perceived as effective by the ED team’s senior leadership, members of its CE team, and by its funders, but if and why this was the case was unclear. We conducted a qualitative case study of the ED Program’s approach to CE to identify and critically examine its components, and to explain whether and how these efforts contributed to the support received by stakeholders.

**Methodology/Principal Findings:**

In-depth semi-structured interviews were conducted with 24 participants with a range of experiences and perspectives related to the ED Program’s CE activities. Our analytic approach combined techniques of grounded theory and qualitative description. The ED Program’s approach to CE reflected four foundational features: 1) enabling conditions; 2) leadership; 3) core commitments and guiding values; and 4) formative social science research. These foundations informed five key operational practices: 1) building the CE team; 2) integrating CE into management practices; 3) discerning the community of stakeholders; 4) establishing and maintaining a presence in the community; and 5) socializing the technology and research strategy. We also demonstrate how these practices contributed to stakeholders’ willingness to support the trials.

**Conclusions/Significance:**

Our case study has identified, and explained the functional relationships among, the critical features of the ED Program’s approach to CE. It has also illuminated how these features were meaningful to stakeholders and contributed to garnering support within the host communities for the open-release trials. Our findings reveal how translating ethical intentions into effective action is more socially complex than is currently reflected in the CE literature. Because our case study delineates the critical features of the ED Program’s approach to CE, it can serve as a framework for other programs to follow when designing their own strategies. And because the findings outline a theory of change for CE, it can also serve as a starting point for developing an evaluation framework for CE.

## Author Summary

Community Engagement (CE) is gaining increasing recognition as an important dimension of biomedical, public health and global health research, including neglected tropical disease research. Yet, there has been little agreement about the specific goals of CE and about the best ways to design, conduct, and evaluate it. CE was an integral component of the ED Program’s initial open release trials in Queensland, Australia and our retrospective case study of their approach to CE in that setting helps explain why the ED program was well accepted in the host communities of the initial field trials. Our analysis has generated an empirically grounded framework for CE that explains the linkages between the foundational ethical and practical commitments made by the ED program and its funders and the specific operational practices adopted by the ED program, through which these commitments were realized. Our case study also identified how these practices were meaningful to stakeholders and contributed to their willingness to support the ED program, in general, and the open-release trials in particular. This framework provides research funders, investigators, implementation partners, policy makers, research ethics committees and host communities an example to follow when designing their CE strategies and assessing whether CE is being conducted to high ethical standards.

## Introduction

The WHO has described dengue as “the most important mosquito-borne viral disease in the world” [1]. Worldwide, more than 2.5 billion people in more than 100 countries are at risk and up to 390 million infections are acquired every year [2, 3]. There are no approved dengue-specific treatments or vaccines and in recent decades there has been a steady increase in the incidence of new infections and the number of countries experiencing outbreaks [2]. New and sustainable approaches to dengue control are desperately needed.

One proposed new approach to controlling dengue is being developed through the Eliminate Dengue (ED) Program. In 2008, McMeniman et al. demonstrated that *Wolbachia*, a bacterium that naturally infects a wide range of insect species, could stably infect *Aedes aegypti* mosquitoes—the main vector of dengue transmission [4]. *Wolbachia* compromises mosquitoes’ disease-vector capacities and has been recently shown to have anti-dengue virus properties [5]. This research laid the foundation for the ED Program, and in the years since, it has had considerable success transitioning the technology from the lab into field-testing, including open-release trials. These scientific milestones have resulted in extensive interest from endemic countries around the world and an ambitious plan for rolling out the technology [Personal communication, Professor Scott O’Neill, May 5, 2014].

A key component of the ED Program’s early open-release trials was community engagement (CE) with stakeholders in the host communities. An initial funding application for support from the Bill & Melinda Gates Foundation’s (BMGF) Grand Challenges in Global Health initiative required a plan to address the social aspects of the technology, including open-release trials. At the time these trials were being planned for Queensland, Australia, there was a growing international controversy about some of the first open-release trials of genetically modified (GM) mosquitoes in the Cayman Islands. These trials met with widespread criticism that emphasized the lack of public and community engagement and elevated these issues to the editorial pages of top scientific journals [6, 7]. These circumstances contributed to the ED team’s emphasis on CE in the development of the *Wolbachia* technology and were important motivators for the funders and the ED team to ensure their approach to CE was well executed.

CE is gaining increasing attention as a dimension of biomedical, public health, and global health research—including vector control research; however, there has been little agreement about the specific goals of CE and about the best ways to design, conduct, and evaluate it [8, 9, 10, 11, 12, 13, 14, 15] In part, this explains the vagueness of the critiques of the Cayman trials and also meant that there was no “off the shelf” guidance for the ED Program to develop their CE strategy. Because of our own team’s previous experience with CE in the testing of other new vector control technologies [10,16], with conducting case studies on CE in various global health research contexts [9,17,18], and our recent work to clarify the ethical goals of CE [8] we were asked to conduct a retrospective case study of the ED Program’s approach to CE in its first open-release trials in communities around Cairns, Queensland, Australia.

Our case study had two purposes: first, to describe and conduct a critical analysis of the CE efforts undertaken by the ED Program leading up to and during the open-release trials in Queensland; and second, drawing from a range of stakeholder perspectives, to explain whether and how these efforts contributed to the support and cooperation received from the host communities. Our analysis delineates a framework for CE that identifies and explains the linkages between the foundational ethical and practical commitments and the specific operational practices adopted by the ED program through which these commitments were realized.

## Methods

We conducted a retrospective qualitative case study informed by grounded theory, an approach we have used successfully in other CE case studies [9,17,18]. The case study focused on a series of open-release trials that began in January 2011 in Yorkey’s Knob and Gordonvale—two communities outside Cairns, Queensland, in northern Australia, and the first two host communities in which the ED Program was launched. The St. Michael’s Hospital Research Ethics Board approved our study.

The goal of sampling in qualitative studies is not to construct a sample that mirrors major demographic features of the target population, but rather to identify key informants with unique experiences and personal knowledge of the phenomenon in question who can provide useful descriptions, insights and explanations of events relevant to the research questions. Two sampling frames were relevant for our research aims: *internal stakeholders* were individuals who were officially employed by the ED program or were involved in its oversight as funders or advisors; and *external stakeholders* were individuals who were engaged by the ED Program either as residents of the host communities, as local or national leaders and/or representatives of local organizations, or both. Guided by a purposeful theoretical sampling strategy [19], recruitment involved working with the ED team to identify internal and external stakeholders with a range of experiences and perspectives related to the ED Program’s CE activities.

A total of 25 individuals were approached and 24 individuals agreed to participate. The 12 internal stakeholders included entomologists, social scientists, CE specialists, and members of the ED team’s senior leadership and communication teams. Our sample of internal stakeholders involved representation from all of the official roles on the ED team that were involved in CE. The 12 external stakeholders included local residents from the trial communities, local and national politicians, representatives from local public health and social service organizations as well as private industry. External stakeholders often had multiple levels of engagement with the ED Program—e.g., both as residents and as representatives of local organizations and businesses. Including external stakeholders in our sample allowed us to deepen our critical perspective on the ED Program’s approach to CE by allowing us to understand what was meaningful to stakeholders outside the ED Program. All participants gave written informed consent.

In keeping with qualitative methodology, data collection and analysis were done in parallel [19, 20]. JVL conducted in-depth semi-structured interviews with each participant. All interviews were video and audio recorded. Recruitment and participation took place in three rounds. An initial round of 11 interviews was conducted in Australia in September 2013. A second round of 11 interviews was conducted in Australia in March 2014, and two final interviews with 2 internal stakeholders were conducted during the ED annual network meeting in Vietnam in May 2014. In keeping with the Grounded Theory method, which encourages the use of multiple forms of data as sources of relevant insights and concepts that might help explain the study findings, we also examined two published papers describing the ED Program’s social research program [21,22].

The initial interviews explored several key conceptual domains, including: internal and external stakeholders’ general observations and insights concerning CE during open-release trials; whether an underlying ‘philosophy’ of CE was apparent; the specific CE activities and practices used; perceived outcomes of these activities and practices; challenges encountered; perspectives on the nature and quality of the ED Program’s engagement efforts; as well as internal stakeholders’ insights regarding the planning, design and management of CE activities by the ED team. To avoid imposing conceptual framings onto participants’ experiences and perspectives, and to facilitate the stakeholders’ own narratives, these domains were used as a guide to prompt and probe stakeholders’, rather than asking them to respond to a set of pre-defined questions from the interviewer’s perspective, as occurs in the administration of a questionnaire. The overarching goal is to elicit the *respondent’s* perspective and concepts, not to seek validation of the interviewer’s conceptualizations.

During the second and third round of interviews, the central concepts and themes developed during the initial analysis were explored in greater depth from the perspectives of both internal and external stakeholders, including the ethical dimensions of the ED Program’s approach to CE, and the meaning of involvement in the CE activities. We made efforts to elicit concerns and any sources of skepticism or negative views during both rounds of interviews and through our sampling decisions. Interviews averaged 50 minutes. Audio recordings of interviews were transcribed verbatim and accuracy checks performed.

The analytic approach combined techniques of grounded theory and qualitative description [19, 23, 24]. Two main rationales informed our choice of method: First, grounded theory emphasizes the experiences of participants, the meaning of these experiences to participants and their understanding of events [25], as opposed to seeking confirmation of investigators’ hypotheses. Second, the grounded theory method aims to generate a theory of the phenomenon in question. The goal is to produce an explanatory account that combines rich description of process with explanations of how various actions and structures lead to specific outcomes of interest [26].

The three authors coded the written transcripts to identify key concepts, categories, and patterns [19, 23, 24, 26]. A constant comparative approach was used to compare findings within and across interviews and between categories [19, 26]. Techniques for ensuring analytic rigor and trustworthiness included comparison of coding between analysts, seeking alternative explanations for the data, and interrogating the coherence of interpretations through deliberations among the analysts [24].

## Results

Our analysis identified several critical dimensions of the ED Program’s approach to CE that help to explain its role in the support ED received from the host communities. These dimensions include the various decisions, practices, and organizational processes employed by the ED team to structure their approach to CE as well as the various ethical commitments realized through their approach. In practice, the ED Program’s approach to CE was not formalized, nor was it overtly planned *a priori*, and the underlying ethical commitments were not articulated explicitly. The aim of our case study was to generate insights about what was done, and why it generated the results it did in an effort to make the underlying assumptions and actions explicit. This allowed us to delineate a framework for CE that can assist others in the planning, conduct, and evaluation of their own CE strategies.

In part one of our results, we have organized our findings into a framework for CE involving two dimensions: (1) the foundations of the ED Program’s CE strategy; and (2) the operationalization of these foundations in the day-to-day conduct of the ED Program. In part two of our results, we demonstrate how the ED Program’s approach to CE was meaningful to external stakeholders.

### CE Framework: Foundations

We identified four features that provided the foundation for the ED Program’s approach to CE: (1) *enabling conditions*; (2) *internal leadership—*including leadership demonstrated by the ED senior management team who had to defend and champion the investment and commitment to CE; (3) *core commitments* and *guiding values* that informed how the ED Program’s approach to CE was operationalized; and (4) *formative social science research* that provided key insights about the local context and about how stakeholders in the host communities wished to be engaged by the ED program.

#### Enabling conditions

There were three enabling conditions that facilitated the ED team’s capacity to “do” CE in a robust way: (1) support from sponsors; (2) regulatory approvals; and (3) an independent risk assessment. Although the funders did not impose specific requirements for CE on the ED Program, the need for, and importance of, CE in the ED Program was consistently and continually encouraged, supported, and reinforced by the Senior Program Officers from the BMGF and the Foundation for the National Institutes of Health (FNIH) who were responsible for managing the BMGF’s investments in the ED Program. The funders recognized that supporting CE should be a priority, based on prior examples of field trials of other modified-mosquito technologies that had failed because of inadequate or inappropriate public engagement processes.

We all knew, the applicants knew, everyone in the program knew how important this was going to be because we all had heard stories about similar types of technology that were inhibited, were stopped, because the public engagement process was not conducted appropriately… (IV3-24, Funder)

The *Wolbachia* technology did not fit neatly into any of the existing regulatory and risk assessment pathways in Australia. The lack of clarity about the appropriate regulatory review processes might have made it easier for the ED Program to deploy *Wolbachia* mosquitoes with minimal regulatory scrutiny. But, instead, the ED Program used the opportunity to engage regulators—an external stakeholder—to clarify an appropriate regulatory pathway and review process for the open-release studies. Additionally, the ED Program’s leadership chose to obtain an independent, comprehensive, expert risk assessment from a respected organization in Australia [27, 28].

Okay, the mosquito feeds on humans so humans are going to be a part of the mix. And that immediately made it very different to a process for releasing a new species into Australia, because in the context of biological control, you’re usually releasing something which is going to feed on a plant or another insect, not a mosquito that’s going to feed on humans. So, the risk assessment processes that you go through for the introduction of a new species into Australia is completely silent on pretty well anything other than environmental impact risk. … I said to [the ED PI], “well this has got to be bigger than that. We really do need to look at this as an all hazards risk assessment and we need to work out what all those hazards are and categorize them.” We just had this huge brainstorming exercise that delivered well over 100 potential hazards. … There were political issues. There were social issues. There were economic issues and there were regulatory issues. And so, we thought well okay, we need a way of tackling this. And again, there’s no blueprint. Nobody has done this in Australia as far as Australian legislation was concerned. (IB2-22: Independent Risk Analyst)

#### Internal leadership

The championing of CE by the ED Program’s senior leadership team, including its Principal Investigator, established CE as a high priority and sent a strong signal to the ED team that CE was to be taken very seriously.

But I think amongst some of the scientists involved there was a view that what science does is so self-evidently a public good that you really don’t need to pay much attention to these issues. . . Equally, and I think this is really important, there were signs of a very different attitude …I don’t think there’s any doubt that [the PI], who was the leading light in this, or a leading light. It was very obvious to me early on that he certainly was well aware of the need to genuinely consider the social aspects. Not simply as a PR exercise. (IB2-18 ED Social Scientist)

Leadership on CE was manifested in three key program decisions: (1) CE would be an integral component of the ED Program; (2) sufficient human and financial resources would be invested in CE; and (3) sufficient time would be dedicated to CE before, during, and after the planned open-release trials. These decisions set the stage for the success of the ED Program’s approach to CE. Table 1 presents eight additional specific features of the ED Program leadership that had a formative role in the design and execution of their CE strategy.

**Table 1:** Demonstrations of leadership by PI and senior program management.

1. Recognized personal responsibility for the design and execution of the CE strategy
2. Dedicated personal time to CE activities, including a wide range of community-facing activities
3. Articulated a clear commitment to conducting CE at a high level of quality to the ED team and to external stakeholders
4. Articulated expectations for how all ED staff should interact with stakeholders and the general public
5. Demonstrated an understanding of, and appreciation for, the importance of context, including the local significance of the “cane toad” biological control program in Queensland, and earlier controversies related to CE in other mosquito release programs, and how these might shape the initial attitudes of the host communities
6. Treated CE as a legitimate central management issue, and not as a peripheral task to be delegated to other parts of the program
7. Trusted experts from social science disciplines with significant responsibility in the context of a very large and complex basic and applied science consortium
8. Provided sufficient resources to CE (time, expertise, funds)

#### Core commitments and guiding values

Our analysis of interviews with internal and external stakeholders identified a clear set of core commitments and guiding values. Although these were never formalized by the ED program, they were explicitly and implicitly evident in the ED Program’s approach to CE, and in the experiences of both internal and external stakeholders. The central idea anchoring the ED Program’s approach was that throughout the entire duration of the project—including the planning and conduct of any open-release trials—*CE had to be "meaningful" to stakeholders themselves* and not simply useful to the investigators. That CE had to be meaningful to stakeholders was embodied and manifested in nine core commitments and guiding values that: (1) served as the ethical foundation for shaping the ED team’s approach to CE; (2) informed how the ED team would interact with stakeholders; and (3) provided practical guidance in terms of designing and conducting specific CE activities and practices. Table 2 presents an in-depth overview of these core commitments and guiding values.

**Table 2:** Core commitments and guiding values.

**Core Commitment or Guiding Value**	**Meaning**	**Supporting Quotation**
**The technology would not be “foisted” on the community**	The host communities had to be supportive of the releases otherwise the open release trials would not proceed.	And I think that was a principle that we had right from the beginning; it wasn’t enough just to have government support, if we didn’t have community support, we weren’t going to foist it on anybody and being very open about that from the beginning”. (IM1-8 Senior Leadership)
		“We need community support for this trial to go ahead, so by you saying no that’s fine, that’s a big part of it. If there’s not enough support in the community, we won’t go ahead with it”. (IC1-01 CE Officer)
**An inclusive view of who constitutes a stakeholder would be adopted.**	The ED team considered “everyone a stakeholder” including residents of the host communities, residents of neighbouring communities, local business owners, media outlets, community-based organizations, public officials, and public health organizations involved in dengue control. This commitment recognized that many people’s interests might be affected by the introduction of a new technology and assigned equal value to these interests. It also meant that the ED team willingly reached out to people and groups whom they believed might oppose the project.	“Stakeholders are anyone who might be interested, impacted, anything to do with our research, our project. It’s quite an extensive list. The field trial community is an example of a stakeholder, and they would be the people who are living within that field site. Within that, you’ve got your participants who are stakeholders, so they are people who are actually involved in the research. Bring it out a little bit and you’ve got environmental groups, business groups, your more common stakeholders that are around, the internal stakeholders, which may be your other dengue control groups or your staff”. (IC1-1 CE Officer)
		“Anyone that this could possibly affect or touch. That sounds big but it really was as big as that. I don’t think we had any limitations on who the stakeholders could possibly be. We prioritized. I think I remember writing lists, lines and lines and lines of people, and then you prioritized as need be.” (IB2-21 Senior Leadership Team)
**The ED team would make themselves available to stakeholders**	Throughout the duration of the project, the ED team “put themselves into the community” by making themselves readily available to stakeholders and actively seeking opportunities to engage.	“We walked the streets of Gordonvale. I remember walking. [The ED PI] walked them, the scientists walked them. We did up beautiful pamphlets. We put them in the letter boxes, every single door to door letter box. We had community engagement, welcome to the community at the local pubs, at the local areas. Sometimes nobody showed up. Sometimes a school teacher showed up and took a bunch of pamphlets because she wanted to talk to the school about it.” (IB2-21 Senior Leadership Team)
		“… I think it’s also being available for people to come and talk to you. It comes in many different forms, from essentially sitting on the side of the street somewhere so if people walk past they can come and talk to you and ask you questions. It’s having information around, and it’s making connections with different people within the community that might operate in networks themselves … By you being known to them, you’re bringing the community to you.” (IC1-01 CE Officer)
**Stakeholders would be listened to and their perspectives would be taken seriously**	Listening was the main dynamic of the interactions between the ED team and the full range of stakeholders and entailed providing opportunities for members of the ED team to hear the perspectives of stakeholders and to learn about their interests. Listening activities were also the basis for ensuring that the community was comfortable with both the scientific and engagement activities.	“You know, we’re always listening. We always want to hear and make sure that … somebody new comes along with a new set of eyes and a new set of questions. And we continue to be responding to what the community is raising or what they might have heard from somewhere else….” (IM1-11 Communications Personnel)
		“… if someone or one person has asked that question, we make sure that that person gets one-on-one feedback. And it is just not a matter of sending an email and a phone call. Often, say, a community engagement officer in Cairns will go back to that person, in-person, and say, look, I have put this to the scientists. And here is more information you might like to read.” [IB2-21 Senior Leadership Team]
**The ED program would be responsive to stakeholder’s questions and concerns.**	The commitment to be responsive meant that the ED team took the interests of stakeholders seriously, and were willing to engage with them, rather than using CE superficially to placate stakeholders.	“If there had been some instances where people went ‘oh we don’t want this’, or, ‘what, you’re just going to come and do this work?’ then the alarm bells went off and we took it very seriously. You’d have to go ‘okay, how else can we connect with these people?’ ‘Do we need to slow things down a little bit?’ ‘Do we need to spend longer explaining it before we get up to the field trial part of releasing?’” (IC1-01 CE Officer)
**Stakeholders would have the opportunity to shape the way they were engaged.**	As part of their commitment to listening and being responsive, from the outset of the project, stakeholders’ perspectives were sought regarding how they preferred to be engaged and the informational content of the engagement activities.	“We got into much more in the way of conversations with people:. . . Wanting to find how out how people wanted to be engaged—what was the methodology that they were most comfortable with? How would they want us to interact with them? Do they really want to participate or do they just want to be informed?” (IB2-18 Social Scientist)
		“Well we were interested in trying to work out what people wanted to do and let’s just dial in, let’s not assume not anything, let’s listen to what people want and how much involvement people might want in the research.” (IM1-08 Senior Leadership)
**Opportunities for dialogue and deliberation among stakeholders and members of the ED team would be created**	As part of their engagement activities, the ED team incorporated explicit opportunities for dialogue and deliberation among stakeholders as well as between stakeholders and members of the ED team, including the program’s leadership. These opportunities allowed disagreement to be expressed, concerns to be raised, and provided opportunities for the ED team to publically offer rationales for their decisions.	“I guess one of the things is that in a world where we talk about branding and marketing so much, both of which are very important, I think perhaps what I’m suggesting is that this is moving away from that kind of approach to trying to open up a genuine dialogue, rather than trying to market the project”. (IB2-18 Social Scientist)
**Respect**	As a core value, respect guided the nature of the relationships between stakeholders and the ED team. Demonstrating respect meant that all stakeholders were treated as people first and foremost and as people whose interests mattered for any decisions related the program. Respect also entailed a commitment to facilitate understanding of the science and to ensure that stakeholders were not placed “under pressure” to decide whether to participate in the activities or to support the research. Explicit permission was also sought to conduct various entomological activities in people’s homes and/or their properties, including the release of mosquitoes on their properties or in the public spaces near their homes.	“Everyone we came into contact with, you treated equally, with respect, with honesty, and genuine. And any concerns they brought or any suggestions, they were all taken on as equally important, I think”. (IB2-21 Senior Leadership)
**Transparency & Honesty**	As guiding values, transparency and honesty meant that the “nothing [was] hidden” from the host communities. The vision of the program was shared and the communities were continually informed of the ED program’s activities and progress—including its successes, failures, and the challenges encountered.	“And thinking that it was really important that we come across as really straight up and honest; we don’t hide anything, we’re just transparent, we tell it as it is and where we wanted to go. And hoping people will see that it might be worthwhile trying to get there and then prepare to take on a little bit of risk and go with us. And so I think that sort of was fundamentally at the core of what I was trying to do.” (IM1-8 Senior Leadership)

#### Formative research

In response to the funders’ requirement to address the social dimensions of the research in the initial grant application, the ED Program’s senior leadership specified that extensive formative social science research would be conducted in advance of initiating engagement activities in the host communities and in advance of any open-release trials. They engaged a local social scientist to design and manage a program of research with four goals: (1) to identify and understand stakeholders’ perspectives towards the proposed *Wolbachia* technology; (2) to understand stakeholders’ perspectives on the history of biological control in the region, and the current sociopolitical context; (3) to gain an understanding of stakeholders’ knowledge of dengue-transmitting mosquitoes and their behaviour; and (4) to identify what these stakeholders wanted to learn more about, and believed others would want to learn more about, and how they wanted to be engaged—including opportunities for becoming involved in the project [21, 22]. From the initiation of the formative research program up to, and beyond, the initial open-release trials, the social scientists engaged stakeholders—including residents of the host communities—using in-depth interviews, focus groups, and written surveys [21].

…[we wanted] to find out how people wanted to be engaged. What was the methodology that they were most comfortable with? How would they want us to interact with them? What level of engagement were they most comfortable with? Do they really want to participate, or do they just want to be informed? (IB2-18 Social Scientist)

The results of this formative research helped the ED team know “what they were up against” and helped identify and clarify what they needed to incorporate into their approach to CE and the design of the open-release trials. For example, the research revealed salient understandings and assumptions of stakeholders regarding dengue, such as the persistent misconception that local “swamp mosquitoes” were dengue transmitters, and concerns about the possibility of another “cane toad disaster” based on stakeholders’ previous experience with biological control that lead to undesirable outcomes in the region [15,16]. The ED team also identified that stakeholders wanted opportunities to become involved in the project as well as opportunities to engage with other stakeholders to learn about their perspectives towards *Wolbachia* [15]. Through these formative research activities the ED Program began to refine and operationalize its core commitments and guiding values, which shaped all of their subsequent interactions and relationships with stakeholders.

### CE Framework: Operations

Our analyses identified five key practices that were adopted by the ED Program to operationalize the foundational ethical and practical commitments described above. These “operational practices” were: (1) building the CE team; (2) integrating CE into internal program management; (3) discerning the community of stakeholders; (4) establishing and maintaining a presence in the host communities; and (5) socializing the technology and the research process.

#### Building the CE team

A dedicated CE team was an integral dimension of the ED Program’s operations. As described above, at the outset of the project, a social scientist was hired who had the expertise to take primary responsibility for designing and conducting the formative research and to guide the translation of the insights gained into a broader engagement plan to be used within the host communities. As the formative research began to take the ED Program increasingly into public view, and as the science progressed towards preparations for open-release studies, dedicated CE officers were hired to work in each host community. Often, the CE officers were residents of the host communities already and their familiarity with the local settings facilitated their engagement with residents.

Alongside the dedicated CE officers, as the planning of specific CE activities was initiated, there was growing recognition that “everyone” on the ED team “does CE” to some extent. This included senior ED Program leadership, field technicians, entomologists, other scientists, as well as staff involved in project communications. For example, scientists were paired with a CE officer to give presentations and answer questions from the public as part of town halls and other public events. Field technicians played a significant role as part of the “public face of the trial” because of their day-to-day interactions with residents who agreed to participate in entomological activities. And the communications team facilitated the development of public relations materials for use in CE—including sharing what was learned through CE within the host communities on the ED Program’s website for wider distribution to other stakeholders. Investment in CE training for ED staff was a key operational priority.

#### Integrating CE into internal program management

The senior leadership of the ED Program recognized CE as a central management issue and ensured that it was incorporated into their day-to-day management priorities. Two main practices were involved. The first entailed having CE Officers provide updates during weekly program meetings, including feedback they had received from field technicians and any relevant insights gained through their interactions with stakeholders.

. . . having the community engagement person in the room as part of these weekly planning meetings that we had, so that there was the rapid feedback. What we were hearing from the communities via our community engagement person was integrated into our operations: Were we releasing too many mosquitoes in some areas of the community? Was there this informal chatter amongst the community that maybe we were getting to the point where people might not be all that happy to participate. . . . So having that rapid turnaround of feedback … was operationally very reassuring to know that we weren’t getting off track with our relationship … we want people out there measuring, listening, and bringing those feelings back into the project in a timely fashion. (IM1-09 Senior leadership)

The second practice entailed each CE officer maintaining a database tracking their interactions with stakeholders. This record-keeping facilitated the ED Program’s commitment to listen and be responsive. For example, if a resident declined participation in the release activities, this was noted in the database. When subsequent release activities were being planned this documentation allowed the ED team to avoid re-contacting this household for permission (recognition that ‘no’ meant ‘no’). Instead, the team would reach out and inform the resident of a new upcoming release, acknowledge their prior response, and seek their perspective on this latest release. Although the databases held a valuable dataset and helped the ED team fulfill their core commitments, the CE team members who used the databases reported that they could likely serve other useful purposes as well, but had not yet been employed more systematically across the program.

#### Discerning the community of stakeholders

The ED team adopted an inclusive view of stakeholders (what we have referred to in our sampling strategy as *external* stakeholders)—i.e. the broad range of individuals, groups, and organizations they believed could be “affected” by their activities at all levels—local, state, and national—and committed to “reaching everyone”. As one ED team member described, “lines and lines” of public officials, individuals, community groups, and organizations were generated and plans put in place to engage them. Discerning the community of stakeholders was not a linear process, but rather a complex and recursive set of activities. Identifying potential stakeholders often began through interactions with public officials because they were seen to be legitimate representatives of relevant constituencies, and provided reliable introductions to other “well connected and respected” local leaders and those with “standing” in the community. As part of their commitment to inclusivity, the ED team consistently reached out to individuals and groups whom they, or others, believed might support the ED Program as well as those who might oppose it. The ED team also made efforts to identify and tap into existing social networks as ways to improve the “reach” of their engagement efforts and to acknowledge and respect the social structures that already existed in the host communities.

My feeling is quite often when anybody walks into a community, it doesn’t matter if it’s dengue work or. . . whatever you’re doing, there are already incredible networks that exist within a community. And if you bypass them and think you know better by coming in your way, then you are potentially really putting the community aside. And there are already, also, really good people within the community building those networks. So, it also is another way of respecting the social structures that already exist and talking to them and connecting to their networks as well.” (IB2-15 CE Officer)

#### Establishing and maintaining a presence in the community

Establishing and maintaining a presence in the community reflected the ED Program’s ongoing commitment to “put themselves into the community” and to be transparent about their work. The goal was to make the ED Program visible and familiar and therefore further facilitate their commitment to make themselves available to stakeholders:

. . . I think it’s also being available for people to come and talk to you. It comes in many different forms, from essentially sitting on the side of the street somewhere so if people walk past they can come and talk to you and ask you questions. It’s having information around, and it’s making connections with different people within the community. . . By you being known to them, you’re bringing the community to you. (IC1-01 CE Officer)

Specific practices included traditional public relations strategies such as disseminating information about the program through pamphlets and posters in public places. They also employed branding practices such as ensuring staff uniforms and vehicles were marked with the ED logo. Other examples included renting a store-front in the Cairns city centre and having various members of the ED team, including the PI, “walk the streets” to interact with residents. Having ED team members accessible in these community settings and at times that were convenient for local residents increased the frequency and richness of interactions and contributed to the team’s commitments to listen and to be responsive to stakeholders’ concerns.

#### Socializing the technology and the research process

The ED team made extensive efforts to socialize the *Wolbachia* technology and their proposed research strategy. This process involved introducing the scientific concepts underlying the technology, explaining what they hoped to achieve through the program, and eliciting host community input on these aims as well as feedback about their CE practices. Numerous individual and group-based activities were undertaken including formal meetings with public officials and local business leaders, town halls, presentations and meetings with community groups, community organizations and local business groups including residents’ associations, the local boating association, Meals on Wheels, the State Emergency Service, and the local Cane Growers Association. Other activities included presentations at area elementary schools, distributing newsletters to interested residents, hosting booths and installments at local community events and festivals, as well as regular “door knocks” at the homes of residents in the release areas—particularly during the open-release trials.

Each of these activities enabled the ED Program to operationalize several core commitments and guiding values including making themselves available to stakeholders, listening and responding to stakeholders’ concerns, and facilitating dialogue.

… if somebody says “you’re releasing a lot of mosquitoes” or “there’s too many mosquitoes around my house. I don’t want to be involved any more”. We follow up with a call. We say “when are you being bitten?” If they’re being bitten at night time, they’re not dengue mosquitoes. They’re not mosquitoes we’re releasing. So we tend to put our trap in the house. …we’ll provide that information back to the resident and say “hey, we’ve done some trapping. Yeah, the mosquito numbers are high but they’re mosquitoes that bite you at night. They’re not the ones we’re releasing.” So we try to educate them that it’s not just our mosquitoes, there’s more than one species. (IM1-10 Senior Leadership)

The ED team also chose to undertake several experiments—“direct experiments”—in response to questions raised by community members about the safety of the technology that were not originally part of the ED Program’s research plan:

We undertook certain research even though we felt as scientists that a lot of the concerns, when you looked at the scientific literature and what we knew about the bacterium, they weren’t really big problems in our mind but mindful of the community felt that they were issues… For example, could *Wolbachia* be transferred to people? So we did a bunch of experiments around that and showed that well no, it couldn’t. We already felt comfortable about that as scientists because lots of mosquitoes have *Wolbachia* that bite people all the time and we see no untoward effect from that. But we wanted to do the actual research to show the people in the community that there wasn’t a problem there and we ended up using that to show regulators as well subsequently. (IM1-08 Senior Leadership)

The results of these experiments were shared with the communities during their engagement activities.

### Support from External Stakeholders

One of the ED program’s more complex commitments was to not “foist” the project on the host communities. Part of socializing the technology involved seeking permission from hundreds of individual households to participate in the ED program’s entomological and/or release activities. When households did not consent to have mosquitoes released on their property, the ED team also refrained from releasing mosquitoes on their neighbours’ adjacent properties, and offered to place mosquito traps at their property. Although residents appreciated these gestures, tensions occasionally arose between the ED Program’s commitment not to foist the technology on the community, and the wishes of individuals who refused to permit releases on their property.

It’s always the fact that we need so much support or we need community support for this trial to go ahead, so by you saying no that’s fine, that’s a big part of it. If there’s not enough support in the community, we won’t go ahead with it. That’s a tricky thing because what is support and how do you measure that? For us, we measured it by the number of people who would sign up and by the fact that there wasn’t strong backlash or opposition when you were giving presentations or out on the street (IC1-1, CE Officer).

In this second section of our results, we identify how the ED Program’s approach to CE was meaningful to stakeholders, even amongst those who were apprehensive, and contributed to their willingness to support the ED Program.

Our analysis of external stakeholders’ accounts of their interactions with the ED team, and internal stakeholders’ reports of these interactions, identified that the ED team encountered apprehension in some stakeholders, and that support varied among individual stakeholders from toleration, to formal institutional collaborations, to various forms of cooperation.

#### Apprehension/opposition

Several internal and external stakeholders recounted their own, and others’ apprehension and opposition to the *Wolbachia* technology, a perspective that was identified predominantly, though not exclusively, in the initial phases of engagement.

I wanted an ultimate feeling of safety, that it wasn’t another bad introduction. If you remember, the cane toad was introduced at Gordonvale and has been an environmental disaster. I said sometimes the scientists get it wrong, so I wanted to be sure. (IC2-12 Local Resident)

The reasons reported for apprehension and opposition were diverse and included unfamiliarity with the technology, difficulty grasping the logic of releasing more (*Wolbachia*-infected) mosquitoes to reduce the overall mosquito population, concerns over potential detrimental effects on both human and environmental health, doubts about whether scientists should be “messing with the balance of nature”, and concerns related to previous experiences with biological control strategies in the region.

What they talked about was bacterial cures and that sort of thing. People became a little bit apprehensive about that, a little concerned, because we had never heard of this sort of thing before. The reception was a little vague, no one really knew much about it IC1-05, Local Resident).

Some residents who refused to permit releases of mosquitoes on their property expressed frustration that the ED team’s policy of not releasing on adjacent properties, and even placing traps on their own properties in an effort to limit their exposure, ultimately meant that they were “just going to release them anyway”, and proceed with the program, despite their individual objections.

#### From apprehension to support from stakeholders: How CE was meaningful to stakeholders

Although some individuals experienced some apprehension, and some residents refused to grant permission for releases on their property, these attitudes primarily reflect resistance to, or discomfort with, the concept of biological control, or the seeming paradox of releasing mosquitoes to control dengue, a mosquito-borne disease, or with the perceived inevitability of the program proceeding, even in the face of individual refusals to cooperate. We encountered no complaints from external stakeholders about the manner in which they were engaged. In fact, external stakeholders often described their experience of the intensive personal engagement with the ED program as instrumental in acknowledging and addressing their concerns, and in winning their confidence and support for the ED program. In their accounts of their experiences with the ED Program, stakeholders believed that they were listened to, that their concerns were taken seriously, and that they were respected as people. External stakeholders also indicated that they valued how ED team members made themselves readily available to them, as well as how they were transparent and honest about their work.

Upon going onto Google and seeing that *Wolbachia* was a derivative from the tsetse fly, I had concerns that there were enough safeguards in the program, that we wouldn’t go from being sore and sick from dengue to being zombies from *Wolbachia*. The JCU team then invited me up to see the research facilities in Cairns, and I went there and they allayed all my fears and questions about introducing this. Once I understood what was being proposed and what was going to go on, I supported it and encouraged it, and wherever possible I went with them to public consultation meetings and encouraged people not to have any great fears because I’d seen what they were doing”. (IC2-12 Local Resident)

And another

The group that was working with us here, they were absolutely brilliant in the way they went about it. They consulted to a degree that I have never seen before or since with the community. They came to the major organizations in our community, they came to the residents’ association. I believe they went to the school … We have a Festival of the Knob, they set up booths there and they set it up on consecutive years. They came to the State Emergency Service and updated us on everything. (IC1-06 Local Resident)

#### Toleration, collaboration and cooperation: Stakeholder investment in the ED program

Although our study was not designed to provide quantitative estimates of the levels of investment by stakeholders, our analysis suggests that the majority of residents in the host communities were “tolerators” of the ED Program—i.e., they agreed that the trials should proceed, they were content with the level and quality of engagement, but showed little interest in taking on any active role beyond regular interactions with the ED team. Our analysis suggests that an attitude of toleration towards the ED Program simply reflects the limits of people’s willingness to invest time and energy in activities that fall outside their normal scope of activities.

And despite extensive investments in time on “education” and communications, there was also some predictable lack of understanding about the technology itself, and the logic of population replacement as a strategy to reduce dengue transmission:

Some people are very supportive but I don’t know if they understand what we’re doing as well, and that’s something that is a difficult one because without that level of understanding you always question. Can they really support it without getting it? You can have really good conversations with people and tell them all about it and they’ll be like yeah, yeah, and then at the end they’re like good luck killing the mosquitoes, I hope you get rid of them. You think, oh, you didn’t understand it. (IC1-01 CE Officer)

Several institutional and organizational stakeholders entered into formal collaborations with the ED team and several individual stakeholders were also willing to actively cooperate with the ED team to perform tasks such as “door-knocking”, which helped to facilitate the work of the ED Program. Queensland Health (QH) and James Cook University (JCU) were the two primary institutions that agreed to collaborate with the ED team. QH and JCU are prominent and respected institutions in the region. They facilitated the entry of the ED team into the host communities through their endorsement of the ED Program, introductions to community leaders, and insight into the host communities.

Individual stakeholders who were willing to cooperate with the ED team included local and national politicians, local community and business leaders, members of community-based organizations, and individual residents. Stakeholders who explicitly cooperated with the ED team also facilitated access to their social networks and thereby assisted the ED team to fulfill their commitment to “reach everyone” and their efforts to socialize the technology. Cooperators participated in community events, spread the word about the ED Program within their social networks, distributed public relations materials, and assisted with enrollment activities. Cooperation was also expressed through the willingness of individuals to allow entomological and release activities to occur in their homes and on their properties. The willingness to cooperate is reflected in the following account.

They took the time to explain it in a way that was very clear to me. And, they explained what they were trying to achieve, where they thought they were at, and what the plan was going forward. And, because of that reason, I could see that our SES [State Emergency Service] group could certainly provide some assistance to them, and I was so enthusiastic about the project that I was able to talk my team into helping them out pretty easily. (IC1-06 Local Resident)

## Discussion

Our case study has identified the critical features of the ED Program’s approach to CE and illuminated how these features were meaningful to stakeholders and contributed to garnering their support for the open-release trials, although it is likely that other less obvious factors also contributed to this support. From the outset, the ED leadership recognized that CE could not simply be a set of practices to facilitate the introduction of the *Wolbachia* technology, but had to be concerned with, and attentive to, *the meaning of CE* as well as *the meaning of the technology* and *the way in which it was being introduced*, to a wide range of stakeholders in the host communities.

The implications of these findings are important in the current climate of increased attention to CE in the field of health research [29, 30, 31] and in vector control in particular [12, 13, 14, 21, 22]. They encourage a new, broader view of CE, which emphasizes its legitimacy as a set of sensibilities, commitments, and ongoing practices that reflect sensitivity to the fundamentally human and ethical dimensions of CE [8]. Further, by elucidating the foundational elements underlying the ED program’s approach to CE, and the ways in which they were operationalized at the host community level, we make explicit connections that have been under-represented and largely implicit in prior discussions about the operational aspects of CE. To date, guidance has focused on specific events (e.g., town hall meetings), delivering key messages to stakeholders, and/or implementing mechanisms to help researchers manage their interactions with stakeholders such as Community Advisory Boards (CABs)[32]. Our findings also highlight how the ways in which core commitments and ethical intentions are translated into action and preserved through relationships with stakeholders appears to be a far more active and complex challenge than has yet been recognized in the CE literature.

Another salient finding was the importance of explicit requirements for CE and sustained material support for CE by the funding agencies. Research funding programs have had an important impact on CE over the years. For example, the U.S. National Institute of Allergy and Infectious Diseases (NIAID) of the National Institutes of Health (NIH) has required Community Advisory Boards (CABs) as a condition of funding for HIV clinical trials for more than 20 years [33], and other funding agencies, such as the U.K.’s Wellcome Trust, have made important contributions to the evolution of CE practices through dedicated research funding programs [34]. Taking the interests of external stakeholders seriously is not a cost-neutral proposition and one of the key lessons in the ED CE case is how the research funders’ decision to support a robust approach to CE contributed directly to the realization of ethical, as well as practical, outcomes. In this case, the BMGF and the FNIH requirements and resources allowed the ED program to titrate the features and intensity of its CE strategy to the real-time demands of its external stakeholders and opened the door for more sustained and intensive approaches to CE in global health. Considerable work remains to be done to develop appropriate strategies to cost these activities and assess their cost-effectiveness, and to determine the appropriate scope and scale of CE required for any given application. But our findings should help clarify the core value proposition of CE, and reinforce the central importance of relationships for CE that is meaningful to external stakeholders.

The ED Program’s approach to CE also involved several important ethical dimensions, expressed through their core commitments and guiding values, and realized through the operational practices of discerning the research community, establishing and maintaining a presence, and socializing the technology and research process. These dimensions align well with some of our own recent work to articulate the ethical goals of CE, in particular, to demonstrate respect for stakeholders as people, first and foremost, and not simply as a means through which investigators can achieve their scientific goals.[8] The ED Program also used their ongoing opportunities for listening to identify and understand the risks of the research as perceived by stakeholders and took steps to respond to these risks in ways that were meaningful to a wide range of stakeholders—for example, by conducting, and reporting back the results of their “direct experiments”. They also created opportunities for dialogue, whereby people could express specific concerns or general disagreement with the program and researchers could justify their actions to stakeholders. These opportunities enhanced the legitimacy of the research.

The ED Program’s attention to ethical dimensions of CE is significant, given the lack of guidance about CE available to the ED team at the time these trials were initiated. The ED team’s commitment to being “straight-up and honest” in all of their interactions with stakeholders contrasts sharply with the secrecy that surrounded the Cayman trials [6] and reflects their recognition that doing so is necessary for the ED Program to garner the type of public support that will be necessary for the *Wolbachia* technology to have a major impact on dengue globally. Further, the ED Program’s explicit commitment not to foist the project on the community is unusual and commendable in a field that continues to wrestle with what it means to obtain the support of the host community and to establish the role of research subjects and citizens in determining whether vector control research should proceed [12, 13, 14]. In the end, “foisting” the technology on some individuals who refused is inevitable with this type of technology, particularly in the research phases when the technology is not being deployed under the authority of an agency with a legislated public health mandate. What obligations are owed to those individuals within a release site who refuse to accept releases on their property remains an open question and one that is likely to become more complicated during larger scale releases. Under these circumstances, concerns about the legitimacy of the project are likely to dominate CE efforts, especially if the technologies or programs are in any way controversial.

All of the stakeholders we interviewed seemed to have a clear sense that the ED team members were not simply “going through the motions”, but were reflecting genuine beliefs about the way stakeholders should be treated. This suggests that specific forms of interaction with stakeholders without the same underlying ethical commitments could be perceived as empty by stakeholders and might jeopardize the process of securing support. As a cautionary note, our findings do not imply that *all* of the ED team’s interactions with stakeholders were effective, or successful, or were perceived as such by stakeholders. But we did not find any examples of serious concerns by any stakeholders that went unaddressed by the ED team. Given the detail of the insights offered by interviewees and the extensive social networks represented by our individual participants, we view this as a salient finding.

The empirically grounded CE framework we have delineated here also offers opportunities for paying greater attention to the relationship between CE operations and important ethical and practical outcomes associated with CE. Our case study provides some promising insights into the inter-personal and social underpinnings of “support” or “acceptance” for community-level interventions. Funders should appreciate the importance of these insights and should undertake efforts to improve their ability to characterize and cost CE activities so that they might be better integrated as a legitimate element of their funding portfolios.

Our study has three significant limitations. First, because of its retrospective nature, participants’ accounts were vulnerable to normal limits of recall. Second, the fact that the open-release trials were conducted with no detected adverse outcomes for the community might have limited deeper consideration by some stakeholders and resulted in insufficiently critical positive attitudes towards the ED Program and the CE approach. And, third, we had to rely on the ED team to help us identify participants, since there were no natural sampling frames outside the ED program to identify external stakeholders. As a result, our sampling strategy was constrained and vulnerable to some selection bias. In particular, it was very easy to identify external stakeholders who held positive views, and reported positive experiences about the ED program, but it was more challenging to find “tolerators” or individuals who opposed the project throughout its duration. We attempted to minimize this bias by: (1) purposively sampling external stakeholders who had been skeptical of the technology at some point in the research process; (2) probing ED team members about any specific external stakeholder concerns and about the nature and range of external stakeholder attitudes they had encountered; and (3) with all participants, delving more deeply into any concerns expressed about the technology and/or the engagement process. These approaches kept us cognizant of the potential for positive framing effects and elicited important insights about external stakeholder concerns and opposition, which helped to minimize its impact. However, it is likely that additional interviews, particularly with more “tolerators” would have revealed important details that are not adequately represented in our findings.

The framework we delineate in our results provides an explanatory account of how and why the ED Program’s approach to CE worked in the Queensland trials. Given that it delineates both the foundational and operational dimensions of CE that contribute to a coherent and ethical CE strategy, the framework outlined here has several potential applications. In the immediate term, it can serve as an example for other teams to follow when designing and planning their own CE strategies, whether engaging in their own open release trials or introducing other biotechnologies, or in any complex stakeholder engagement. This is not to suggest that ‘one-size-fits-all’, as each setting and intervention will have its own unique characteristics and challenges, and some settings will necessitate more or less intense CE strategies than the one reported here. It also provides funders, partner institutions, regulators, research ethics committees, civil society organizations, and the general public with a means to begin to assess whether CE is being conducted to high ethical standards. In the longer-term, the ED CE architecture might serve as a useful starting point for developing a robust evaluation framework that could be applied prospectively to guide the planning and implementation of CE strategies, and retrospectively to evaluate their associated processes and outcomes [35, 36].
